# Supplementary material for: Preliminary Study and Observation of “Kalamata PDO” Extra Virgin Olive Oil, in the Messinia Region, Southwest of Peloponnese (Greece)
Source: Foods. 2019 Nov 23;8(12):610. doi: 10.3390/foods8120610 (PMC6963909; doi:10.3390/foods8120610)
Supplement: Supplementary file 1 [file foods-08-00610-s001.pdf]

foods

MDPI

Supplementary data

Preliminary Study and Observation of "Kalamata PDO" Extra Virgin Olive Oil, in the Messinia Region, Southwest of Peloponnese (Greece)

Supplementary Table 2. Influence of the extraction method on the sterolic profile

Sterols and triterpene diols	Mean ±SD	2-phase<br>Mean ±SD	3-phase<br>Mean ±SD	Difference<br>P-value
Cholesterol (%)	0.11 ±0.03	0.10±0,03	0.11±0,04	n.s
24-methylene-cholesterol %	0.32±0.09	0.33±0,10	0.32±0,07	n.s
Campesterol %	3.71±0.38	3.77±0,43	3.61±0,24	n.s
Campestanol %	0.05±0.03	0.06±0.03	0.04±0.02	0.007
Stigmasterol %	0.74±0.19	0.78±0.21	0.67±0.14	0.019
Chlerosterol %	0.85±0.07	0.84±0.07	0.85±0.08	n.s
β-Sitosterol %	80.73±3.73	80.42±3,94	81.21±3.45	n.s
Sitostanol %	0.37±0.30	0.44±0.37	0.26±0.08	0.011
Δ-5-avenasterol %	12.28±3.96	12.34±4,34	12.21±3.37	n.s
Δ-5, 24-stigm/dienol %	0.29± 0.10	0.29±0.11	0.28±0.09	n.s
Δ-7-stigmastenol %	0.19±0.09	0.19±0.06	0.20±0.12	n.s
Δ-7-avenasterol %	0.28±0.11	0.31±0.12	0.24±0.08	0.008
Apparent b-Sitosterol %	94.63±1.07	94.55±1.33	94.76±0.45	n.s
Total Erythrodiol %	2.85±1.25	2.86±1.01	2.83±1.59	n.s
Total sterols (mg/kg)	1033.3±150.1	1005.2±156.8	1077±130.1	n.s

Results are expressed as mean ± standard deviation (SD). Abbreviation, n.s =non-significant. Differences between means of 2-phase vs 3-phase centrifugal system were tested for statistical significance using analysis of variance (ANOVA). Statistical significance level was set at $P < 0.05$.

Supplementary Table 2. Influence of the extraction method on wax esters content

Wax Esters (mg/kg)	Mean ±SD	2-phase<br>Mean ±SD	3-phase<br>Mean ±SD	Difference<br>P-value
Wax Esters C40-C46(WEs)	67.20±18.88	64.09±15.6	72.05±22.57	n.s
Wax Esters C42-C46 (TWEs)	28.38±9.62	28.87±9.07	27.61±10.61	n.s

Results are expressed as mean ± standard deviation (SD). Abbreviation, n.s =non-significant. Differences between means of 2-phase vs 3-phase centrifugal system were tested for statistical significance using analysis of variance (ANOVA). Statistical significance level was set at $P < 0.05$.
